# Circulating MIF, D-DT, and Soluble CD74 in End-Stage Heart Failure Patients Receiving LVAD: An Exploratory Clinical Study and Effects on Adult Cardiac Myofibroblasts

**DOI:** 10.3390/biomedicines14051031

**Published:** 2026-04-30

**Authors:** Maxim Kunze, Moritz Uhlig, Alexander Theißen, Christian Stoppe, Christian Beckers, Jan Larmann, Rachad Zayat, Ajay Moza, Jürgen Bernhagen, Andreas Goetzenich, Christian Bleilevens, Josefin Soppert

**Affiliations:** 1 Department of Anesthesiology, Faculty of Medicine, University Hospital RWTH Aachen, 52074 Aachen, Germany; 2 Department of Anesthesiology, Intensive Care, Emergency and Pain Medicine, University Hospital Würzburg, 97080 Würzburg, Germany; 3 Department of Cardiac Anesthesiology and Intensive Care Medicine, Deutsches Herzzentrum der Charité, 13353 Berlin, Germany; 4 Charité-Universitätsmedizin Berlin, Corporate Member of Freie Universität Berlin, Humboldt-Universität zu Berlin, 10117 Berlin, Germany; 5 DZHK (German Center for Cardiovascular Research), Partner Site Berlin, 10785 Berlin, Germany; 6 Department of Cardiac Surgery, Faculty of Medicine, University Hospital RWTH Aachen, 52074 Aachen, Germany; 7 Division of Vascular Biology, Institute for Stroke and Dementia Research (ISD), LMU University Hospital, Ludwig Maximilian University (LMU) Munich, 81377 Munich, Germany; 8 German Centre for Cardiovascular Research (DZHK), Partner Site Munich Heart Alliance, 80636 Munich, Germany; 9 Munich Cluster for Systems Neurology (SyNergy), 81377 Munich, Germany; 10 Medical Affairs, Abiomed GmbH, 52074 Aachen, Germany

**Keywords:** macrophage migration inhibitory factor, MIF, soluble CD74, D-dopachrome-tautomerase, D-DT, cardiac fibroblasts, heart failure, end-stage heart failure, ESHF, LVAD

## Abstract

**Background:** Emerging evidence highlights the importance of the MIF–sCD74 axis in health and disease, including its role in regulating cell death. While studies in routine cardiac surgery suggest perioperative relevance, its role in end-stage heart failure (ESFH) patients undergoing left ventricular assist device (LVAD) implantation remains unexplored. Moreover, although MIF and sCD74 induce necroptosis in neonatal cardiac myofibroblasts, the effects of MIF, its paralog D-DT, and sCD74 on adult cardiac myofibroblasts (CMFs) are unknown. **Methods:** Plasma concentrations of sCD74, MIF and D-DT were measured perioperatively in a small cohort of patients with ESHF undergoing LVAD implantation (n = 20). As a preclinical model of ESHF, primary adult CMFs were treated with recombinant MIF, D-DT and sCD74 to evaluate their effects on cellular viability and health. **Results:** In LVAD patients, sCD74 and D-DT levels were significantly increased 24 h postoperatively, whereas MIF levels were reduced compared to baseline. ROC curve analysis demonstrated a good discriminatory power of 24 h post-OP sCD74 (AUC = 0.83), sCD74/MIF ratio (AUC = 0.82), and D-DT levels (AUC = 0.88) for acute kidney injury, composite outcome, and right heart failure (RHF), respectively. In adult CMFs, MIF and sCD74 synergistically reduced viable cell counts (*p* = 0.0083), whereas D-DT reduced cell counts in an sCD74-independent manner (*p* = 0.0004). Yet, measures of metabolism, proliferation, apoptosis and necrosis along with inflammatory gene expression remained unchanged. **Conclusions:** Our findings indicate that the balance of MIF, D-DT, and sCD74 during LVAD implantation may be clinically relevant. In particular, an imbalance characterized by elevated sCD74 or D-DT and reduced MIF levels 24 h post-surgery was associated with unfavorable clinical outcomes. Yet, the current findings are exploratory and hypothesis-generating because of a small sample size. Thus, the prognostic value of plasma levels for postoperative complications after LVAD implantation, and the effects of MIF/D-DT/sCD74 imbalance on cardiac myofibroblasts, need to be validated in larger cohorts and in advanced human experimental models.

## 1. Introduction

Despite medical advances, cardiovascular diseases (CVD) remain the foremost cause of morbidity and mortality in industrialized societies today [1,2]. Among these, coronary heart disease (CHD) represents the most prevalent entity with numerous patients suffering from complications following ischemic myocardial infarction (MI) such as acute kidney injury and sepsis [1,3]. Inflammatory processes triggered by ischemic myocardial infarction initiate essential cardiac remodeling, leading to scar formation and healing [4]. However, when inflammation becomes dysregulated and persistent, it drives excessive fibrosis and adverse remodeling [5]. These structural aberrations impair cardiac function and may progress to heart failure (HF), the terminal disease stage associated with increased mortality [5,6]. Among inflammatory mediators of CVD, macrophage migration inhibitory factor (MIF) and its paralog D-dopachrome tautomerase (D-DT, respectively MIF-2) have emerged as pivotal players [7,8,9]. MIF was shown to play a key role in innate as well as adaptive immunity [10,11,12]. In recent years, its role in CVD has been the focus of increasing research interest. MIF is expressed by a variety of immune and non-immune cells, including cardiomyocytes (CM) and cardiac fibroblasts (CF) [13]. While MIF has been implicated in inflammatory pathologies such as atherosclerosis and MI [9,12,14,15,16], emerging evidence highlights its context-dependent dual function, with protective roles in acute phases and disease-exacerbating effects in chronic or persistent activation states [17,18,19].

Both MIF and D-DT bind to the type II transmembrane protein CD74 [20,21], the plasma membrane form of the invariant chain of MHC class II (major histocompatibility complex class II) [22], which has emerged as the cognate MIF receptor and as a key regulator in cardiovascular disease. For intracellular signaling to be initiated, membrane-bound CD74 must form a complex with the co-receptor CD44 [23], but complexation with the MIF chemokine receptors CXCR2 or CXCR4 may also mediate CD74 activation [24]. Through interaction with CD74, MIF or D-DT modulate central processes of MI such as inflammation, immune cell activity, glucose metabolism and tissue remodeling [11,12,25,26]. A soluble form of CD74 (sCD74), generated by proteolytic cleavage [27,28], has been proposed to act as a regulator of MIF signaling, with the potential to mediate both protective as well as pro-inflammatory responses [29].

Clinical studies have shown that MIF and D-DT levels increase intraoperatively in patients undergoing cardiac surgery, whereas sCD74 levels decrease [8]. While intraoperative elevated MIF levels have been associated with a lower risk of atrial fibrillation, elevated D-DT levels have been linked to increased rates of organ failure [8]. The presence of stable MIF/sCD74 complexes is associated with a smaller incidence of acute kidney injury [8]. These findings underscore the clinical relevance of the MIF/sCD74 interaction in acute cardiovascular conditions.

Despite these insights, our understanding of this MIF/sCD74 balance in chronic adverse cardiac remodeling and heart failure remains limited. Thus, in a small, exploratory cohort of patients with end-stage heart failure (ESHF) undergoing left ventricular assist device (LVAD) implantation (n = 20), we analyzed circulating MIF, D-DT and sCD74 levels. Sampling at preoperative, immediate postoperative, and 24 h postoperative time points during LVAD implantation allows for the characterization of MIF, D-DT and sCD74 kinetics, as well as the evaluation of their prognostic significance for postoperative complications in ESHF patients undergoing LVAD implantation.

In hearts from patients with end-stage heart failure (ESHF), differentiated myofibroblasts have been identified as the predominant fibroblast population [30]. In previous work, we demonstrated that the interplay between MIF and its soluble receptor, sCD74, induces neonatal cardiac myofibroblast death and inflammatory responses [29]. However, a comprehensive characterization of the MIF/sCD74 regulatory axis in adult cardiac myofibroblasts (CMFs), a more preclinical relevant model for ESHF, has been lacking. Furthermore, effects of D-DT in interaction with sCD74 are unknown.

Therefore, we investigated the effects of MIF, D-DT and sCD74 on the survival and vitality of adult CMFs in in vitro models for advanced chronic heart failure.

## 2. Materials and Methods

### 2.1. Culture of Murine Cardiac Fibroblasts

Cryopreserved stocks of murine cardiac fibroblasts were obtained from collaborating research teams (Kramann Laboratory working group), which conducted the primary isolation of these cells from adult mouse hearts [31].

Cells were thawed, resuspended and cultured in 75 cm^2^ tissue culture flasks (TPP Techno Plastic Products AG, Trasadingen, Switzerland; Cat. No.: 90075) with a fibroblast growth medium containing DMEM/GlutaMAX (Gibco Thermo Fisher Scientific, Grand Island, NY, USA; Cat. No.: 31966-021), 10% fetal bovine serum (FCS) (Sigma-Aldrich, St. Louis, MO, USA; Cat. No.: F7524) and 1% penicillin/streptomycin (P/S) (10.000 IE/mL) (Sigma Aldrich, St. Louis, MO, USA; Cat. No.: P07881-100 mL). Cell splitting was performed when confluence reached about 90%, using an appropriate ratio. For experiments, cardiac fibroblasts were seeded with a density of 1000 cells per well in 48 well plates (Greiner Bio-One GmbH, Frickenhausen, Germany; Cat. No.: 665180) precoated with fibronectin 0.5 µg/mL (Sigma Aldrich, St. Louis, MO, USA; Cat. No.: F1141-5 MG) and gelatin 0.02% (Carl Roth, Karlsruhe, Germany; Cat. No.: 0646.1) and maintained in a fibroblast growth medium for 3 days.

### 2.2. Experimental Setup

**Figure 1:**
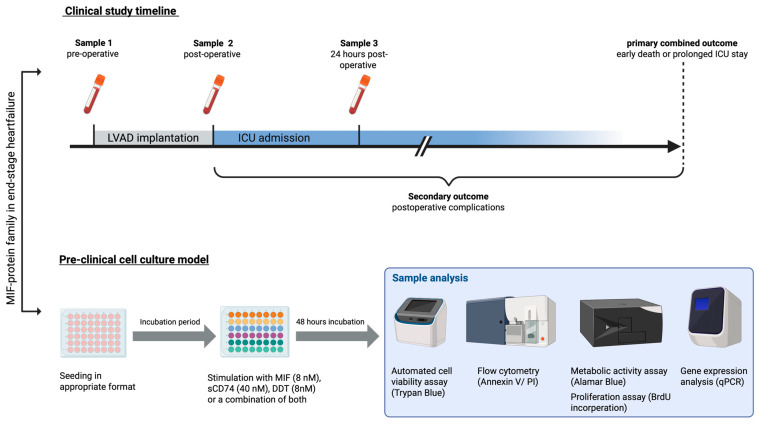
Experimental setup. **Upper panel**: For the clinical exploratory cohort, blood samples from patients end-stage heart failure undergoing left ventricular assist device implantation (LVAD) surgery were taken at baseline (pre-operative), directly at ICU admission (post-operative) and 24 h postoperatively. Plasma levels of MIF, D-DT and sCD74 were measured by ELISA. Primary outcome was defined as early death within the first 30 days or prolonged intensive care unit stay (ICU) with at least 20 days. Secondary outcome postoperative complications, such as acute kidney failure or right heart failure, were assessed. **Lower panel**: As a preclinical in vitro end-stage heart failure model, adult cardiac fibroblasts were seeded in a 48-well plate with a fibroblast growth medium and incubated for 3 days with medium changes every second day. Next cells were treated with either rMIF (8 nM), sCD74 (40 nM), rD-DT (8 nM) or a combination of rMIF/sCD74 or rD-DT/sCD74 in a fibroblast growth medium. In the control group (control), only fibroblast growth medium has been used. After 48 h of incubation, samples were analyzed with Trypan Blue survival assay, Annexin V/PI FACS staining, Alamar Blue metabolic assay, BrdU-incorporation proliferation assay or gene expression with qPCR. Created in BioRender. Bleilevens, C. (2026), https://BioRender.com/8hzk43r.

Cardiac fibroblasts were incubated with a fibroblast growth medium over three days. Afterwards, cells were stimulated with an 8 nM recombinant murine macrophage migration inhibitory factor (rMIF) (Abcam, Cambridge, UK; Cat. No.: ab219128), 40 nM murine soluble CD74 (sCD74) (Cusabio, Houston, TX, USA; Cat. No.: CSB-EP004956MO), 8 nM recombinant D-dopachrome tautomerase (rD-DT) (Novus Biologicals, Wiesbaden-Nordenstadt, Germany; Cat. No.: MBP2-61995-100 µg) or a combination of both (rMIF/sCD74) or (rD-DT/sCD74) for 48 h as described by Soppert et al. (2018) [29]. Control group (Control) cells were incubated with a fresh fibroblast growth medium for the same time. The stimulants were incubated on ice for 30 min and diluted afterwards to the desired concentration with a warm fibroblast growth medium (Figure 1).

For flow cytometry analysis, cells were seeded in a six-well plate precoated with fibronectin/gelatine by adding 100,000 cells/well. On day three after seeding, cells were stimulated with rMIF, sCD74, rMIF/sCD74, rD-DT or rD-DT/sCD74.

To assess cell proliferation using a BrdU-incorporation proliferation assay (Abcam, Cambridge, UK; Cat. No.: ab126556), cells were seeded in a 96-well plate precoated with fibronectin/gelatine by adding 100 µL of cell suspension at a density of 2000 cells/mL.

### 2.3. Alamar Blue Metabolic and Trypan Blue Survival Assays

The metabolic activity of cells was evaluated with Alamar Blue assay. After incubation with 10% Alamar Blue (Bio-Rad, Hercules, CA, USA, Cat. No.: BUF012A) in a growth medium for 4 h and 8 h, metabolization was assessed through colorimetric analyses using Tecan Infinite 200 Pro microplate reader (Tecan, Männedorf, Switzerland) at a dual wavelength of 540/630 nm. The percentage of difference between treated wells and the control was performed according to the manufacturer’s protocol (Table S4).

Cell counts were assessed through staining with Trypan Blue (Sigma Aldrich, St. Louis, MO, USA; Cat. No.: T8154-20 mL). Cells were detached using 100 µL Accutase (Sigma Aldrich, St. Louis, MO, USA; Cat. No.: A6964) per well, and then incubated at 37 °C for 15 min until complete detachment. Following this, 100 µL of a fibroblast growth medium was added into each well. The cells were then transferred into a 1,5 mL reaction tube (Sarstedt AG & CoKG, Nümbrecht, Germany; Cat. No.: 72.690.001) while gently scraping the surface of the well using a 200 µL pipette tip. A total of 20 µL of cell suspension was mixed with an equal amount of a 0.4% Trypan Blue solution and transferred to counting slides (Bio-Rad, Hercules, CA, USA; Cat. No.: 1450015). Total as well as viable cell count were analyzed using an automatic cell counter (Bio-Rad, Hercules, CA, USA; TC20 Automated Cell Counter). To avoid counting of cell debris or clumping, a cell size threshold between 8 µm and 22 µm was chosen (gating). For each condition, technical triplicates were analyzed, and their mean was used for subsequent calculations.

### 2.4. RNA Isolation from Cells

For RNA isolation, cells were cultured in a fibronectin-/gelatin-precoated 6-well plate and stimulated according to the protocol. Cells were incubated for 16 h and lysed with 600 µL RLT lysis buffer (Qiagen, Venlo, The Netherlands; RNeasy Mini Kit, Cat. No.: 74106). Two wells of the same condition have been pooled to reach an appropriate amount of RNA. RNA isolation was performed using the RNeasy Mini Kit and performed according to the manufacturer’s protocol. mRNA quantity and quality were measured using the Nanodrop 2000c (Thermo Fisher Scientific, Waltham, MA, USA). A total of 2000 ng of RNA was reverse-transcribed into cDNA using the High-Capacity cDNA Reverse Transcription Kit (Applied Biosystems, Carlsbad, CA, USA; Cat. No. 4368814) according to the manufacturer’s instructions.

### 2.5. Quantitative Real-Time PCR (qRT-PCR)

qRT-PCR was performed with TaqMan probes (Thermo Fisher Scientific, Waltham, MA, USA; Table S8) using 50 ng of cDNA. Glyceraldehyde-3-phosphate (GAPDH) was used as a housekeeping gene. Using the ΔΔCt method, relative quantity values were calculated and normalized to control.

### 2.6. Immunohistochemical Staining

Cells were plated in a 24-well format and after one, five or seven days of incubation, with medium change every other day, stained for alpha smooth muscle actinin (α-SMA, Thermo Fisher Scientific, Waltham, MA, USA; Cat. No.: 14-9760-82) and Vimentin (Thermo Fisher Scientific, Waltham, MA, USA; Cat. No.: PA5-27231). The medium was discarded and the cells washed once with PBS. Afterwards they were fixated through incubation with Paraformaldehyde 4% for 20 min and then permeabilized with 0.01% Triton (Sigma Aldrich, St. Louis, MO, USA; Cat. No.: T8787-50ML). Between every step the cells have been washed three times with PBS (Gibco Thermo Fisher Scientific, Grand Island, NY, USA; Cat. No.: 14190-094) added with 0.05% Tween20 (Carl Roth, Karlsruhe, Germany, Cat. No.: 9127) (PBS-T). Blocked with 2% bovine serum albumin (BSA) (Sigma Aldrich, St. Louis, MO, USA; Cat. No.: A1470-25G) for an hour, incubation with the primary antibody for α-SMA and Vimentin took place overnight at 4 °C. Afterwards, cells were incubated with the adequate secondary antibodies for another hour. Images were taken with the Keyence BZ-X800 fluorescence microscope (Keyence Corporation, Japan, Osaka).

### 2.7. Flow Cytometry (FACS)

The percentage of necrotic cells after stimulation with rMIF, rD-DT, murine sCD74 and the combination of rMIF/sCD74 and rD-DT/sCD74 was assessed with fluorescence-automated cell sorting (FACS) (BD FACS Canto II laser) using a FITC Annexin V Apoptosis Detection Kit with PI (BioLegend, San Diego, CA, USA, Cat. No.: 640914). Cells were detached on day four after seeding using 1 mL Accutase for 15 min at 37 °C. The cell suspension was washed twice with ice-cold 5 mM EDTA FACS buffer, centrifuged at 300× *g* at 4 °C after each step and afterwards stained according to the manufacturer’s protocol with Annexin V and PI. We evaluated 100,000 detected events per sample. Cells negative for both Annexin V and PI staining were classified as viable. Cells positive for Annexin V but negative for PI were defined as early apoptotic. PI-positive, Annexin V-negative cells were categorized as necrotic, while cells double-positive for Annexin V and PI were considered late apoptotic.

### 2.8. BrdU Cell Proliferation Assay

To further quantify the proliferation rate under different stimuli, a Bromodeoxyuridine (BrdU) cell proliferation assay (Abcam, Cambridge, UK; Cat. No.: ab126556) was performed. The assay has been performed according to the manufacturer’s protocol with an incubation time of 16 h with BrdU starting a day after stimulation. Colorimetric analysis was performed using Tecan Infinite 200 Pro microplate reader (Tecan, Männedorf, Switzerland) at a dual wavelength of 450/550 nm.

### 2.9. Measurement of MIF, D-DT and sCD74 in Plasma

The analysis of patient samples was approved by the local ethics committee RWTH Aachen Germany (EK151/09) and registered at clinicaltrials.gov (NCT 02488876). The study has been performed in accordance with the declaration of Helsinki. Informed consent of all patients was obtained prior to sample collection. Blood samples were taken at baseline (pre-OP), directly at intensive care unit admission (direct post-OP) and 24 h post-surgery as described in earlier studies [32]. Cryopreserved plasma samples were thawed once and analyzed using enzyme-linked immunosorbent assays (ELISA, Helsinki, Finland) for MIF (R&D Systems, Minneapolis, MN, USA; Cat. No: DY289), diluted 1:10, D-DT (AssayGenie, Dublin, Ireland; Cat.No: HUFI00923), diluted 1:2, and sCD74 levels (Sigma Aldrich; St. Louis, MO, USA; Cat. No.: RAB1351-1KT) with a dilution of 1:4. All assays were performed according to the manufacturers protocol. For D-DT and sCD74, absorbance was measured at 450 nm using a Tecan Infinite 200 Pro microplate reader (Tecan, Männedorf, Switzerland). For MIF, absorbance was measured at two wavelengths: 450 nm and 540 nm, and the 540 nm value was subtracted from the 450 nm reading. A standard curve was generated using GraphPad Prism (version 10.4.0, GraphPad Software, Inc., La Jolla, CA, USA) by plotting known concentrations of the standard against their corresponding signal values. Sample concentrations were calculated by interpolation from the standard curve.

### 2.10. Analysis of Patient Data

Plasma MIF, D-DT and sCD74 levels were retrospectively analyzed in patients undergoing left ventricular assist device (LVAD) implantation, a procedure known to induce mechanical stress and trigger sterile inflammation [33,34]. Furthermore, patients with measurable stable MIF/sCD74 complexes in blood had less acute kidney failure after cardiac surgery [8].

Plasma levels of MIF, D-DT and sCD74 were analyzed to assess their potential predictive value for patient recovery, both individually and with relation to age and gender as covariates. Additionally, we calculated the sCD74/MIF and sCD74/D-DT ratio by dividing the plasma concentration of sCD74 by the plasma concentration of MIF and D-DT, respectively. The primary outcome was a composite event, defined as either prolonged intensive care unit (ICU) admission of 20 days or longer or mortality within 30 days after LVAD implantation surgery. To ensure a conservative assessment of patient outcomes by ICU admission, which has been reported to average up to 21 days [35,36], we selected this outcome to reduce potential bias introduced by shortened ICU stays resulting from early mortality.

As a secondary outcome, Receiver Operating Characteristic (ROC) curve analyses were performed using directly postoperative and 24 h postoperative biomarker levels to assess their predictive value for key postoperative complications associated with LVAD surgery, including pneumonia, acute kidney injury (AKI), rethoracotomy, and right heart failure (RHF).

### 2.11. Statistical Analysis

Statistical analysis and graphical presentation of cell culture data were done using GraphPad Prism (version 10.4.0, GraphPad Software, Inc., La Jolla, CA, USA). Outlier detection was performed using Grubbs’ test (*α* = 0.05). Normal distribution was assessed using the Shapiro–Wilk test. For in vitro data, statistical comparisons of normalized values between treatment groups were performed using a one-sample two-sided *t*-test against the theoretical mean of 100% followed by Bonferroni’s correction, calculated in Microsoft Excel (version 16.100.4, Microsoft Cooperation, Redmond, WA, USA) to assess differences relative to the control group. Absolute cell counts were compared by one-way ANOVA with Tukey’s multiple comparisons test. Flow cytometry data was analyzed by two-way ANOVA with post hoc Bonferroni correction. Plasma levels of MIF, D-DT and sCD74 paired across time points were compared using two-way ANOVA (mixed model analysis) utilizing Tukey’s correction for multiple comparisons. Non-parametric data was analyzed with the Kruskal–Wallis test. Plasma levels or their calculated ratio were compared for each time point with Sidak correction for multiple comparisons. Descriptive statistics of patient data were performed using the “dplyr” package (version 1.1.4). Retrospective analysis of patient data was done using R version 4.4.3 (28 February 2025) “Trophy Case” (The R Foundation for Statistical Computing, Vienna, Austria). *p* < 0.05 is considered significant. A comprehensive description of the statistical procedures is available in the Supplementary Materials (see “Exploratory retrospective clinical data”).

## 3. Results

### 3.1. Time Course of Plasma Levels of MIF, D-DT and sCD74

**Figure 2:**
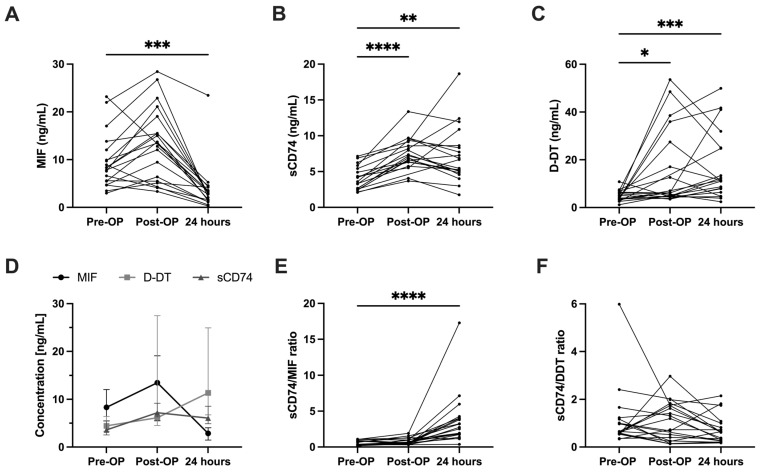
Plasma levels over time. MIF-, sCD74- and D-DT-plasma levels were measured in patients receiving LVAD implantation surgery at baseline (pre-OP) (*n* = 19), directly at ICU admission following the surgery (post-OP) (*n* = 19) and at 24 h postoperatively (24 h) (*n* = 20). (**A**) MIF levels decreased compared to baseline at 24 h after surgery (24 h vs. pre-OP (median (1st; 3rd quartile)): 2.86 (1.47; 4.05) ng/mL vs 8.31 (5.50; 12.05) ng/mL, *p* = 0.0002). (**B**) sCD74 levels increased compared to baseline in the first 24 h after LVAD implantation (post-OP vs. pre-OP: 7.19 (6.34, 8.88) ng/mL vs. 3.59 (2.55, 5.43) ng/mL, *p* < 0.0001; 24 h vs. pre-OP: 6.07 (4.88, 8.56) ng/mL vs. 3.59 (2.55, 5.43) ng/mL, *p* = 0.005) as well as (**C**) D-DT levels (post-OP vs. pre-OP: 6.15 (4.66; 22.29) ng/mL vs. 4.43 (3.42; 6.30) ng/mL, *p* = 0.037; 24 h vs. pre-OP: 11.34 (7.48; 24.83) ng/mL vs. 4.43 (3.42; 6.30) ng/mL, *p* = 0.0007). Panel (**D**) pictures the time course of all three together. (**E**) The calculated sCD74-to-MIF ratio at 24 h postoperatively compared to baseline (24 h post vs. pre: 0.362 (0.317, 0.853) vs. 2.631 (1.739, 4.010; *p* < 0.0001). (**F**) The sCD74-to-D-DT ratio remains equal to baseline through the watched period. *p* < 0.05 is considered significant. Statistics for (**A**–**C**,**E**,**F**): Kruskal–Wallis with Dunn’s multiple comparison test. Statistics for (**D**): Mixed-effects model (repeated measures, matched by time with missing values), followed by Tukey’s multiple comparison test (three comparisons per time point, nine comparisons in total). *p* < 0.05 is considered significant. * *p* < 0.05, ** *p* < 0.01, *** *p* < 0.001, *** *p* < 0.0001.

First, we retrospectively analyzed an exploratory cohort of 20 ESHF patients undergoing LVAD implantation surgery. Clinical baseline parameters are depicted in Table 1. Plasma concentrations of MIF, sCD74 and D-DT were measured at baseline (pre-OP), directly at ICU admission (post-OP) and 24 h later. Compared to baseline, sCD74 plasma levels remain significantly increased until 24 h post-surgery (post-OP vs. pre-OP: 7.19 (6.34, 8.88) ng/mL vs. 3.59 (2.55, 5.43) ng/mL, *p* < 0.0001; 24 h vs. pre-OP: 6.07 (4.88, 8.56) ng/mL vs. 3.59 (2.55, 5.43) ng/mL, *p* = 0.005). MIF levels remain unchanged directly at ICU admission and decrease significantly at 24 h compared to baseline (24 h vs. pre-OP: 2.86 (1.47; 4.05) ng/mL vs. 8.31 (5.50; 12.05) ng/mL, *p* = 0.0002). At baseline, MIF levels exceed sCD74 levels about 2.3-fold. However, this relationship reverses with sCD74 levels surpassing MIF 24 h postoperatively by about 2.1-fold. D-DT levels increase directly (post-OP vs. pre-OP: 6.15 (4.66; 22.29) ng/mL vs. 4.43 (3.42; 6.30) ng/mL, *p* = 0.037) and 24 h after LVAD implantation compared to baseline (24 h vs. pre-OP: 11.34 (7.48; 24.83) ng/mL vs. 4.43 (3.42; 6.30) ng/mL, *p* = 0.0007) (Figure 2A–D).

In addition, we calculated the ratio of sCD74 to MIF as well as sCD74 to D-DT plasma levels. The sCD74-to-MIF ratio increases significantly in the first 24 h after surgery compared to baseline (24 h vs. pre-OP: 2.631 (1.739, 4.010) vs. 0.362 (0.317, 0.853), *p* < 0.0001) (Figure 2E), while the sCD74-to-D-DT ratio remains equal through the first 24 h compared to baseline, as sCD74 and D-DT levels increase in a likewise manner (Figure 2F).

Taken together, during the course of LVAD implantation, the sCD74/MIF ratio reverses with an excess of MIF preoperatively and an excess of sCD74 24 h postoperatively, while the sCD74-to-D-DT ratio remains stable throughout surgery. Intriguingly, the 24 h postoperative excess of sCD74 relative to MIF, along with elevated D-DT levels after LVAD implantation in ESHF patients, resembles experimental conditions in our ESHF cell culture model, where adding recombinant D-DT alone or a combination of high sCD74 and low MIF levels triggered a significant loss of cardiac myofibroblasts.

### 3.2. High sCD74/MIF Ratio and High D-DT Levels at 24 h Indicate Post-Operative Complications

Next, we aimed to investigate whether cytokine levels or their ratios may serve as predictors of postoperative complications commonly associated with LVAD implantation surgery.

**Figure 3:**
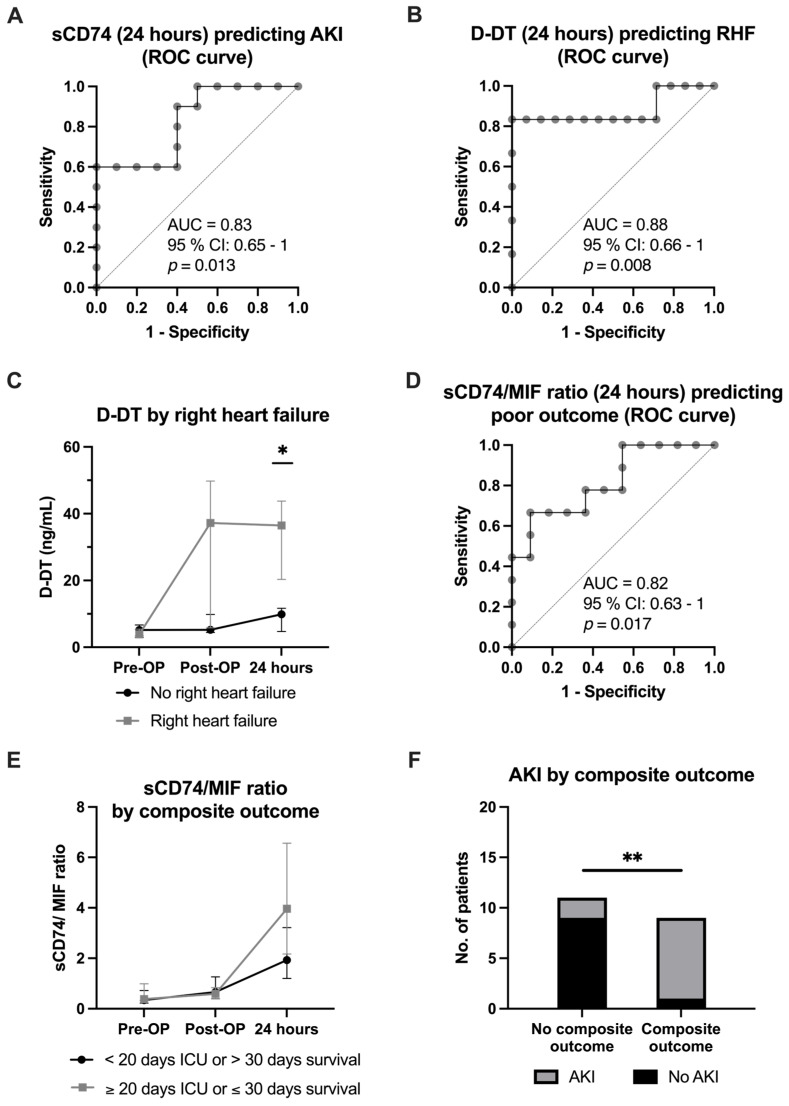
Receiver operating characteristics (ROC) demonstrating discriminatory ability of sCD74, D-DT plasma levels as well as sCD74/MIF ratios at 24 h postoperatively. (**A**) sCD74 levels demonstrate a good discriminatory performance for patients with acute kidney failure (AKI) (AUC = 0.830, 95% CI = 0.647–1.0, *p* = 0.0126) (*n* = 10 out of 20 patients). (**B**) D-DT at 24 h postoperatively shows good discriminatory power to discriminate patients developing right heart failure (RHF) (AUC = 0.88, 95% CI = 0.66–1.0, *p* = 0.008) (*n* = 6 out of 20 patients). (**C**) D-DT plasma levels are significantly elevated 24 h after surgery for LVAD implantation in patients with postoperative right heart failure (RHF) compared to those without (no RHF vs. RHF (median (Q1; Q3)): 9.86 (4.71; 11.70) ng/mL vs. 36.48 (20.34; 43.76) ng/mL, *p* = 0.041). Values represent median with interquartile range (Q1–Q3). (**D**) At 24 h the sCD74/MIF ratio shows a good ability to identify patients reaching the primary combined outcome (AUC = 0.82, 95% CI = 0.63–1.0) (*n* = 9 out of 20 patients). (**E**) Timeline of the calculated sCD74/MIF ratio dichotomized by the composite outcome. The sCD74/MIF ratio is graphed as median with interquartile range (Q1–Q3). (**F**) Eight out of nine patients reaching the primary outcome experienced AKI, while two out of eleven patients not reaching the composite outcome experienced AKI (*p* = 0.0017). Statistics for (**A**,**B**,**D**): Simple logistic regression, unadjusted for covariates. Statistics for (**C**,**E**): Mixed-effects model (repeated measures, matched by time with missing values), followed by Šídák’s multiple comparison test (3 comparisons per time point, 9 comparisons in total). Statistics for (**F**): Chi-Square test. *p* < 0.05 is considered significant. * *p* < 0.05, ** *p* < 0.01.

The discriminatory power of MIF, D-DT, sCD74, the sCD74/MIF ratio and the sCD74/D-DT ratio at each time point was first analyzed to assess their ability to distinguish between patients with and without postoperative complications, including pneumonia, acute kidney injury (AKI), rethoracotomy, right heart failure (RHF), and a composite outcome defined as either death within 30 days after surgery or prolonged ICU stay of at least 20 days (Table S1). The 24 h postoperative sCD74 levels showed good discriminatory power for identifying patients who developed acute kidney injury (AKI), a common postoperative complication (AUC = 0.830, 95% CI = 0.647–1.0, *p* = 0.0126) (Figure 3A). D-DT as well as sCD74/D-DT levels demonstrated good ability to discriminate between patients with and without right heart failure (RHF) directly after the procedure (D-DT: AUC = 0.83, 95% CI = 0.61–1.0, *p* = 0.023, sCD74/D-DT: AUC = 0.83, 95% CI = 0.63–1.0, *p* = 0.023) and at 24 h postoperatively (D-DT: AUC = 0.88, 95% CI = 0.66–1.0, *p* = 0.008; sCD74/D-DT: AUC = 0.87, 95% CI = 0.67–1.0, *p* = 0.011) (Figure 3B). The median D-DT plasma levels were significantly elevated in patients with RHF (no RHF vs. RHF: 9.86 (4.71; 11.70) ng/mL vs. 36.48 (20.34; 43.76) ng/mL, *p* = 0.041) (Figure 3C). At the 24 h point, the sCD74/MIF ratio has a good discriminatory power for the composite outcome (AUC = 0.82, 95% CI = 0.63–1.0, *p* = 0.0017) (Figure 3D). When stratified by the composite outcome, the sCD74/rMIF ratio did not differ significantly at any measured time point (Figure 3E). In contrast, other postoperative complications, such as pneumonia or rethoracotomy, were not associated with any MIF, sCD74 or D-DT concentration or ratio at any time point (Table S1). Notably, 80% of patients with postoperative AKI also experienced a prolonged ICU stay or 30-day mortality (*p* = 0.0017 (Chi-square test)) (Figure 3F).

**Figure 4:**
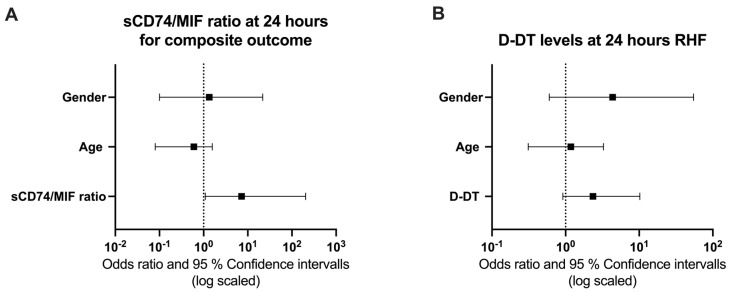
Forest Plots of the sCD74/MIF ratio and D-DT levels at 24 h after LVAD implantation. (**A**) Predictive Firth Regression analysis demonstrates that elevated sCD74/MIF ratios at the 24 h time point were independently associated with the adverse composite outcome (exp(beta) = 7.25, 95% CI = 1.09–205.0, *p* = 0.030). (**B**) Predictive Firth Regression analysis did not confirm the ROC-derived predictive value of D-DT levels at 24 h post-LVAD implantation, either independently or after adjustment for covariates (exp(beta) = 2.36, 95% CI = 0.92–10.2, *p* = 0.077).

Given the promising ROC performance of the sCD74/MIF ratio for the composite outcome, we next assessed the prognostic relevance of the sCD74/MIF ratio for early death in the first 30 days after surgery or prolonged ICU admission with at least 20 days as a composite outcome. We performed a regression analysis including age and gender as relevant covariates. Neither preoperative nor immediately postoperative sCD74/MIF ratios were suitable to predict the composite outcome, either independently or with adjustments for covariates (Figure S1). In contrast, at 24 h post-surgery, a higher sCD74/MIF ratio was independently linked to a worse composite outcome (exp(beta) = 7.25, 95% CI = 1.09–205.0, *p* = 0.030) (Figure 4A, Table 2). However, this association did not remain significant after correction for multiple testing and should therefore be interpreted with caution as exploratory. Cut-point analysis (Youden index) identified an optimal cut point of a sCD74/MIF ratio of 3.817. Kaplan–Meier curves did not show significantly higher probability for remaining in ICU in patients with an elevated sCD74/MIF ratio (>3.817) at 24 h postoperatively (HR = 1.8, 95% CI = 0.63–5.18, *p* = 0.276) (Figure S2).

As ROC curve analysis also showed a good discriminatory power of D-DT levels for RHF, we determined the prognostic relevance of D-DT for RHF by performing regression analysis. D-DT levels independent of time cannot reliably predict post-OP RHF, neither independently nor with adjustments for covariates (Figure S1 and Figure 4B, Table 3). Cut-point analysis (Youden index) identified an optimal cut point of levels of 8.092 ng/mL. Kaplan–Meier curves did not show significantly higher probability for remaining in ICU in patients with an elevated sCD74/MIF ratio (>8.092 ng/mL) at 24 h postoperatively (Figure S2) (HR = 1.95, 95% CI = 0.59–6.48, *p* = 0.273).

Taken together, our explorative findings show that particularly the sCD74/MIF ratio and D-DT levels at 24 h after LVAD implantation may represent a promising biomarker for adverse outcomes, including early mortality/prolonged ICU stay and right heart failure, respectively. While the sCD74/MIF ratio and D-DT levels at 24 h demonstrate good discriminatory power for early mortality/prolonged ICU stay and RHF, respectively, caution is warranted when interpreting these associations. ROC AUC values (e.g., 0.83–0.88) are derived from a small, single-center dataset without adjustment for multiple comparisons. Accordingly, their prognostic utility remains preliminary and requires validation in larger, independent cohorts.

### 3.3. Adult Cardiac Fibroblasts Got Activated to Myofibroblasts by Culture-Associated Handling

**Figure 5:**
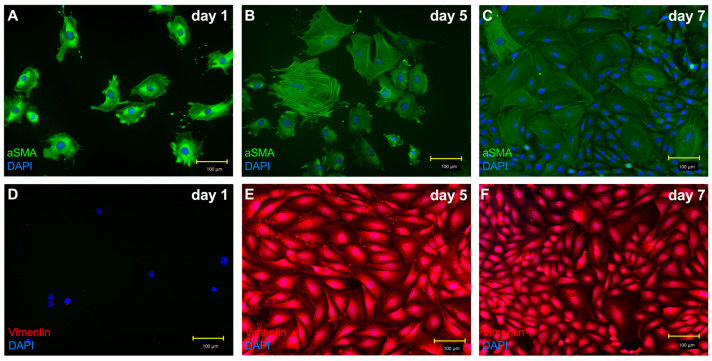
Adult cardiac fibroblasts transdifferentiate into cardiac myofibroblasts after culturing for five days. Cultured adult cardiac fibroblasts express alpha-smooth muscle actin (α-SMA) and vimentin cultured over time adopting a myofibroblast-like phenotype. Adult cardiac fibroblasts were cultured for one (**A**,**D**), five (**B**,**E**) and seven (**C**,**F**) days and afterwards stained through immunohistochemical staining. Panels (**A**–**C**) represent alpha-SMA (green) and DAPI (blue), while (**D**–**F**) demonstrate the expression of vimentin (red) and DAPI (blue). Images were acquired at 20×x magnification.

Based on immunohistochemical expression of alpha-smooth muscle actin (α-SMA) and vimentin protein, the adult cardiac fibroblasts adopted a myofibroblast-like phenotype by day 5 (Figure 5). Thus, experimental stimulation and analysis performed at this point were carried out on activated fibroblasts exhibiting features consistent with myofibroblasts. Therefore, we refer to these cells as myofibroblasts rather than fibroblasts in the following sections.

### 3.4. rMIF/sCD74 and rD-DT/sCD74 Stimulation Decrease Cell Count in Cardiac Myofibroblast Cultures

Three days after seeding, adult murine cardiac myofibroblasts (CMFs) were stimulated with rMIF, sCD74 and a combination of both at concentrations previously reported to be most effective [29]. Subsequently, viable and total cell counts were quantified using an automated cell counter following Trypan Blue staining.

**Figure 6:**
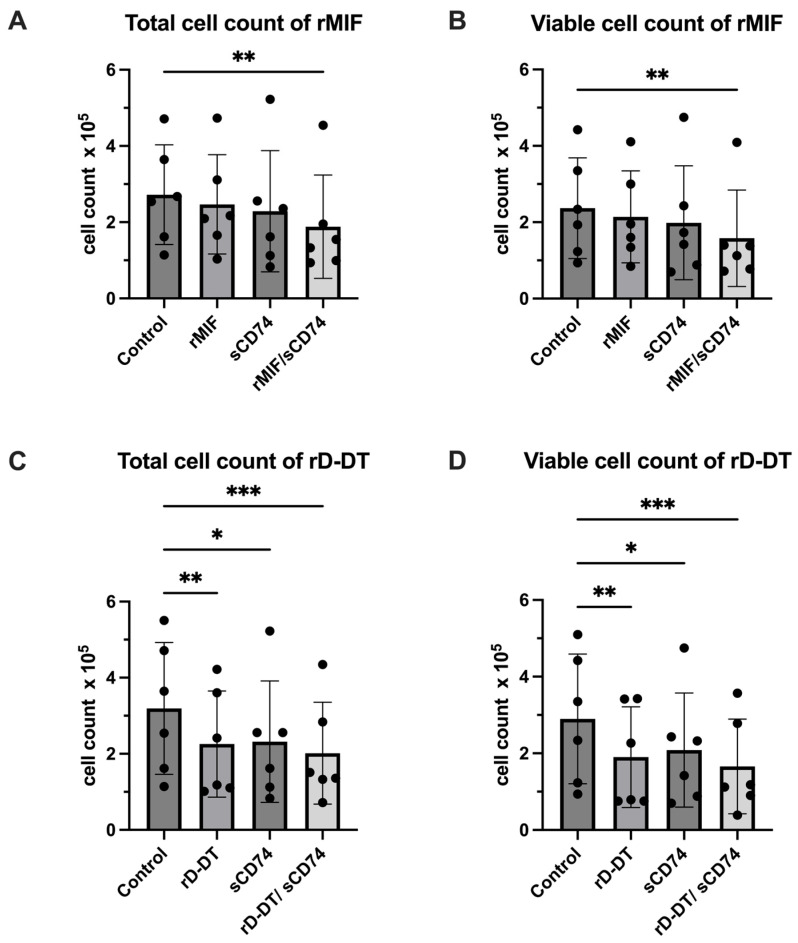
sCD74 in combination with rMIF or rD-DT decreases cell counts in Trypan Blue exclusion assay. Adult cardiac myofibroblasts were stimulated with either 8 nM of rMIF, 40 nM sCD74 and a combination of both or 8 nM rD-DT, 40 nM sCD74 or a combination of both (*n* = 6 per group). Afterwards, Trypan Blue exclusion assay was performed to determine cell survival. In absolute total and viable counts. (**A**,**B**) Total as well as viable cell counts were decreased after co-stimulation with rMIF/sCD74. (**C**,**D**) Either rD-DT alone or rD-DT/sCD74 co-stimulation reduced total and viable cell counts. Cell counts were compared to control using one-way ANOVA with Tukey’s multiple comparisons test. *p* < 0.05 is considered significant. * *p* < 0.05, ** *p* < 0.01, *** *p* < 0.001 (Tables S2 and S3).

In adult CMFs, treatment with rMIF or sCD74 alone did not lead to a significant reduction in viable and total cell counts while co-stimulation of rMIF and sCD74 showed a significant reduction in both counts compared to control (total cell count: control vs. rMIF/sCD74 (mean ± SD) 2.608 ± 1.309 × 10^5.^ vs. 1.885 ± 1.357 × 10^5^; *p* = 0.004; viable cell count: control vs. rMIF/sCD74 (mean ± SD) 2.369 ± 1.319 × 10^5^ vs. 1.581 ± 1.263 × 10^5^; *p* = 0.0083) (Figure 6A,B, Table S2).

Isolated treatment with rD-DT showed a significant decrease in viable and total cell counts in relation to control (total cell count: control vs. rD-DT (mean ± SD) 3.194 ± 1.73 × 10^5.^ vs. 2.256 ± 1.395 × 10^5^; *p* = 0.0077; viable cell count: control vs. rD-DT (mean ± SD) 2.897 ± 1.691 × 10^5^ vs. 1.9202 ± 1.313 × 10^5^; *p* = 0.0004). A similar observation was made for the co-treatment with rD-DT and sCD74 (total cell count: control vs. rD-DT/sCD74 (mean ± SD) 3.194 ± 1.73 × 10^5^ vs. 2.017 ± 1.338 × 10^5^; *p* = 0.0028; viable cell count: control vs. rD-DT/sCD74 (mean ± SD) 2.897 ± 1.691 × 10^5^ vs. 1.657 ± 1.234 × 10^5^; *p* = 0.0004) (Figure 6C,D, Table S3).

**Figure 7:**
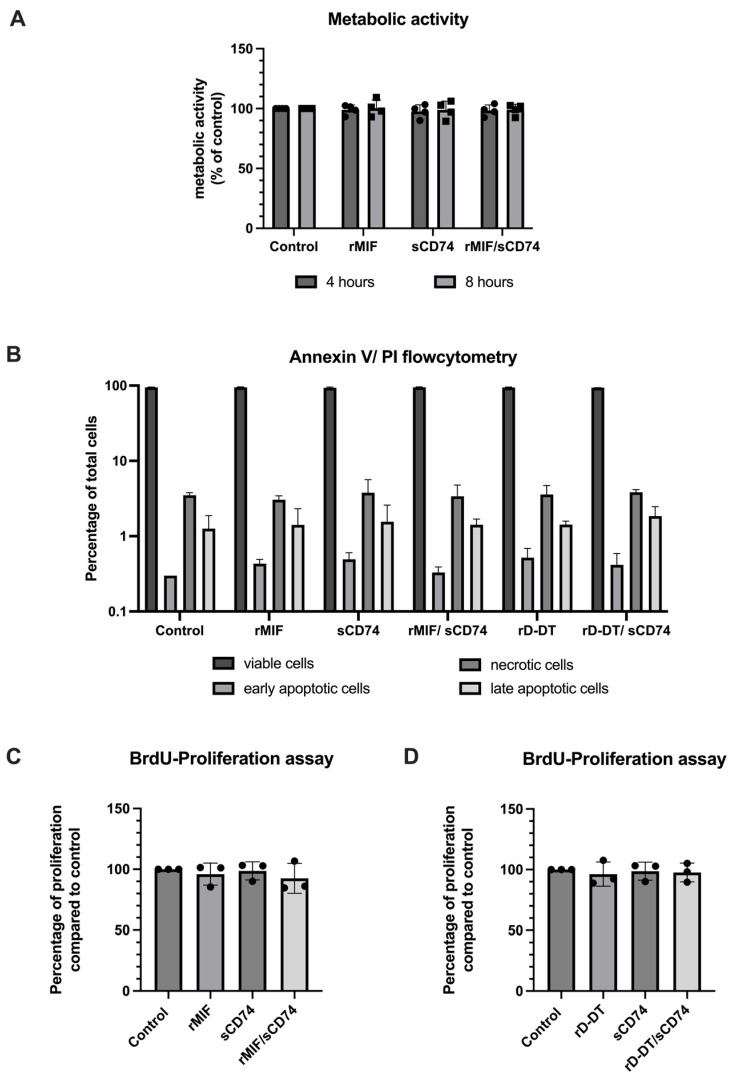
Metabolic activity, apoptotic/necrotic cell proportions and proliferation properties are not altered by treatment. Adult cardiac myofibroblasts were stimulated with rMIF, sCD74, rD-DT or a combination of sCD74 with rMIF or rD-DT for 48 h. (**A**) Via Alamar Blue assay, the metabolic activity was analyzed after four and eight hours showing no significant changes (*n* = 4 per group). (**B**) To assess proportions of early and late apoptotic, necrotic and viable cells, Annexin V/PI stained flowcytometry was used showing equal proportions compared to control under each treatment (*n* = 3 per group). (**C**,**D**) To assess proliferation activity, BrdU was added to the last 16 h of the 48 h stimulation period. Afterwards, BrdU incorporation was analyzed and did not reveal significant differences in cell proliferation rates normalized to control (theoretical 100%) (*n* = 3 per group). Data is shown as mean ± standard deviation. *p* < 0.05 is considered significant. Statistics for (**A**,**C**,**D**): One-sample *t*-test with post hoc Bonferonni correction. Statistics for (**B**): Data analyzed by two-way ANOVA with post hoc Bonferroni correction.

The decrease in cell count was not accompanied by changes in metabolic activity (Figure 7A), apoptotic or necrotic cell death assessed by Annexin V/PI flow cytometry (Figure 7B) or alterations in proliferation (Figure 7C,D). The proportion of viable cells remained comparable across treatments in Annexin V/PI flow cytometry (Figure 7, Tables S4–S6). None of the inflammatory genes investigated were found to be upregulated under any of these conditions (Table S7).

Taken together, MIF and sCD74 act synergistically to affect adult cardiac myofibroblast cell counts, while exogenous D-DT reduces the cell count independently of sCD74.

## 4. Discussion

To our knowledge, this study is the first to assess circulating levels of MIF, sCD74, and D-DT in plasma samples of patients with end-stage heart failure before and after LVAD implantation surgery. Furthermore, we investigated the effects of the atypical cytokines MIF and D-DT and the soluble form of their receptor CD74 on total and viable cell numbers of adult cardiac myofibroblasts in order to extend previous findings from neonatal models into a more translationally relevant context.

In chronic diseases like heart failure, the role of MIF and D-DT seem conflicting. Experimental studies in knockout mice demonstrate that both MIF and D-DT play protective roles, limiting contractile dysfunction and tissue remodeling during pressure overload [7,37,38]. In a human failing heart, MIF expression is often increased, especially in HF due to ischemic cardiomyopathy, while D-DT expression is lowered in advanced stages [7]. In circulation of HF patients, MIF correlates with disease severity and progression [39,40], while reduced D-DT levels are associated with reduced survival [7].

In end-stage heart failure refractory to medical therapy, left ventricular assist devices are used as a therapy option either as a bridge to transplant or as definite therapy [41]. In our exploratory cohort of ESHF patients undergoing LVAD implantation, we observed that MIF levels increase slightly, but not significantly, at ICU admission and significantly declined 24 h postoperatively in the presented exploratory cohort of ESHF patients undergoing LVAD implantation. This temporal pattern is comparable to the MIF profile observed in patients undergoing conventional cardiac surgery with the use of a cardiopulmonary bypass (CPB) [8]. However, in contrast to the peri- and postoperative decrease in sCD74 typically seen in cardiac surgery patients [8], sCD74 levels rose significantly following LVAD implantation. Furthermore, plasma levels of D-DT are increased directly after surgery and stay elevated for the first 24 h, while reported dynamics in cardiac surgery patients show a decline postoperatively [8].

At 24 h postoperatively, sCD74 seems to be associated with increased rates of acute kidney injury (AKI), while D-DT may be associated with the occurrence of right heart failure (RHF). In contrast, MIF did not show an association with any of the post-operative complications commonly observed after LVAD implantation [42,43]. Compared to healthy individuals, patients with acute HF demonstrate elevated plasma levels of D-DT, whereas D-DT expression is downregulated in chronic HF [7].

Previously, in cardiac surgery patients, the presence of stable circulating MIF/sCD74 complexes was associated with improved postoperative outcomes and D-DT levels were associated with increased rates of organ dysfunction [8]. In our study, we did not assess these complexes directly but instead measured absolute concentrations of MIF, sCD74 and D-DT independently. Notably, in the LVAD cohort, we observed a high sCD74 concentration relative to MIF at 24 h postoperatively. This imbalance was associated with an adverse composite outcome, defined as early mortality or a prolonged ICU stay. Given that postoperative complications increase proportionally with the length of ICU stay [44], this observation suggests a potential prognostic value of postoperative sCD74 and MIF plasma levels in identifying patients at risk for adverse events or prolonged critical care following LVAD implantation. Yet, the limited cohort size restricted analysis to individual timepoints, assessed with and without covariant adjustment. Robust model fitting would necessitate larger sample sizes. Consequently, shown results could overoptimize the value of sCD74/MIF ratios and their predictive abilities. Nevertheless, other studies investigating the roles of MIF and sCD74 have highlighted the importance of their balance as a key regulatory mechanism and a potentially valuable prognostic indicator. For example, in patients with decompensated cirrhosis, elevated MIF levels combined with low sCD74 serum concentrations were associated with poorer survival outcomes, likely due to enhanced inflammatory and profibrotic signaling [45]. Similarly, in melanoma patients, a high circulating MIF/sCD74 ratio correlated with reduced survival probability [46]. Notably, Fukuda et al. demonstrate that high levels of sCD74 inhibited melanoma cell proliferation and promoted apoptosis under IFN-γ stimulation by suppressing the MIF/CD74/AKT signaling axis [46]. These findings suggest a shared regulatory mechanism involving MIF and sCD74 across different pathological conditions and cell types.

MIF in combination with sCD74 has previously been shown to induce necroptosis and inflammatory signaling in neonatal cardiac myofibroblasts [29]. However, adult fibroblasts differ markedly from their neonatal counterparts in both gene expression and responsiveness to external stimuli, which may alter their susceptibility to cytokine-mediated injury [47,48,49]. To address this, we examined the impact of cotreatment with recombinant MIF and sCD74 on primary adult cardiac myofibroblasts. Further, we evaluated whether recombinant D-DT, a structural and functional homolog of MIF, which also signals via CD74, exerts similar effects on cell survival.

Trypan Blue exclusion assay revealed reduced cell counts of adult cardiac myofibroblasts following treatment with MIF/sCD74, comparable to previously reported findings in neonatal cardiac myofibroblasts [29]. Further, the effects of MIF’s structural homolog D-DT seems to be independent of sCD74, as D-DT alone reduced total as well as viable cell counts significantly, while co-treatment with sCD74 enhanced these effects. Interestingly, the observed reduced cell counts following MIF/sCD74 or rD-DT treatment were not accompanied by changes in metabolic activity, proliferation or classical cell death. These findings suggest that neither MIF/sCD74 nor D-DT directly induce cell death of adult CMFs under basal culture conditions. Instead, treatment with MIF/sCD74 or D-DT may increase cellular vulnerability to secondary stressors, such as mechanical sheer forces, assay-related physicochemical stress or handling procedures, which could account for the discrepancies observed between experimental readouts. Accordingly, other studies demonstrate that Trypan Blue might result in the immediate rupture of cells leading to a decreased number of cells but without changes in the proportion of live-to-total counts [50,51].

In contrast to our previous findings in neonatal cardiac fibroblasts, where sCD74 in combination with MIF induced upregulated expression of an inflammatory gene involved in microbial and innate defense [29], the expression of these genes appeared largely unaffected by rMIF/sCD74 or D-DT stimulation in adult cardiac myofibroblasts. Since cell counts declined in the absence of type I interferon-inducible gene activation following MIF/sCD74 or D-DT stimulation, our finding suggests that type I interferon-induced genes are not required, at least not in adult cardiac myofibroblasts, to induce higher susceptibility to cell rupture and death.

## 5. Limitations

We recognize that our study has several limitations. While the cohort analyzed in this retrospective study is relatively small, the observed trends provide preliminary evidence and underline the potential relevance of the investigated biomarkers. Further validation in larger, adequately powered studies are required and reported results are limited to a hypothesis-generating character. Larger cohorts are required to fit adequate statistical models.

The withdrawal of the murine sCD74 reagent from the market limited sample sizes and prevented further planned analyses. Future adequately powered studies may uncover additional effects.

## 6. Conclusions

Clinically, in end-stage heart failure patients undergoing LVAD implantation, elevated postoperative sCD74/MIF ratios were associated with early mortality or a prolonged ICU stay, while increased D-DT levels were observed in patients with postoperative right heart failure. However, given the small sample size, limited adjustment, and lack of robustness after correction for multiple testing, these findings should be interpreted with caution and considered exploratory.

In a preclinical cell culture model of adult cardiac myofibroblasts, reduced cell counts were observed under conditions of high sCD74 combined with low MIF, suggesting a potential synergistic effect, whereas D-DT decreased cell counts independently of sCD74. While these findings point toward a possible role of the MIF–sCD74–D-DT axis in modulating cellular responses, the underlying mechanisms remain incompletely understood.

However, whether these effects translate to changes in myofibroblast abundance in human myocardial tissue and subsequently impact myocardial adaptation remains un-clear. These observations are therefore hypothesis-generating, and the potential of MIF, D-DT, and sCD74 as exploratory biomarkers requires validation in larger cohorts as well as further mechanistic investigation.

## Figures and Tables

**Table 1:** Descriptive statistics of LVAD patients. The cohort of patients undergoing LVAD implantation surgery analyzed in this exploratory study consists of 20 individuals, 15 male and 5 female. The median age (IQR) was 67 years (61.25, 73.00) and the median survival has been 2345.5 days (2254.75, 2451.50). Median duration at ICU was 8.38 days (4.00, 23.69). The interquartile range is reported as boarders of the first (Q1) and third (Q3) quartiles.

Analysis Columns	No. of Patients	Percentage	*n*
Sex			
Male	15	75	20
Female	5	25	20
Composite outcome			
ICU ≥ 20 days and/or survival ≤ 30 days	9	45	20
ICU < 20 days and/or survival > 30 days	11	55	20
Risk factors			
Arterial hypertension	13	65	20
Diabetes	7	35	20
COPD	4	20	20
Post-operative complications			
Acute kidney failure	10	50	20
Pneumonia	14	70	20
Right heart failure	6	30	20
Rethoracotomy	7	35	20
	Median	IQR	*n*
Age (years)	67	61.25, 73.00	20
BMI (kg/m^2^)	29.04	26.23, 32.87	20
Postoperative survival (days)	2346.5	2254.75, 2451.50	20
ICU duration (days)	8.38	4.00, 23.69	20
Left ventricular ejection fraction (%)	20	18.00, 24.25	20
INTERMACS Score	4	3, 4	20
EuroSCORE II	11.07	7.32, 15.66	20
HeartMate 2 risk score	1.28	0.73, 1.97	20
MIF plasma levels (ng/mL)			
Baseline	8.31	5.54, 10.98	19
Postoperative	13.46	5.93, 17.28	19
After 24 h	2.86	1.56, 3.72	20
sCD74 plasma levels (ng/mL)			
Baseline	3.59	2.59, 5.17	19
Postoperative	7.19	6.34, 8.88	19
After 24 h	6.07	4.99, 8.40	20
D-DT plasma levels (ng/mL)			
Baseline	4.43	3.42, 6.30	19
Postoperative	6.15	4.66, 22.29	19
After 24 h	11.34	7.48, 24.83	20
sCD74/MIF ratio			
Baseline	0.36	0.32, 0.84	19
Postoperative	0.59	0.44, 0.96	19
After 24 h	2.63	1.74, 3.98	20
sCD74/D-DT ratio			
Baseline	0.65	0.56, 1.09	19
Postoperative	1.20	0.33, 1.75	19
After 24 h	0.62	0.27, 0.86	20

**Table 2:** Regression models for the sCD74/MIF ratio predicting composite outcome. Predictive Firth Regression models demonstrate a predictive value of the sCD74/MIF ratio at 24 h after LVAD implantation surgery—it independently predicted the composite endpoint, defined as early death within 30 days or prolonged ICU stay exceeding 20 days (95% CI = 1.09–205; *p* = 0.03). At earlier time points, the sCD74/MIF ratio—either alone or adjusted for covariates—did not show predictive significance.

Predictive Firth Regression Models for Composite Endpoint				
Characteristic	Exp(Beta)	95% CI	*p*-Value	q (FDR)
Covariates only				
Age	0.72	0.22, 1.61	0.4	
Gender	1.77	0.26, 13.5	0.6	
Preoperative sCD74/MIF ratio				
sCD74/MIF ratio pre-OP	1.87	0.71, 5.71	0.2	0.6
Age	0.59	0.13, 1.47	0.3	
Gender	1.25	0.15, 9.88	0.8	
Postoperative sCD74/MIF ratio				
sCD74/MIF ratio post-OP	1.00	0.39, 2.42	>0.9	>0.9
Age	0.67	0.18, 1.58	0.4	
Gender	1.51	0.22, 11.5	0.7	
24 h sCD74/MIF ratio				
sCD74/MIF ratio 24 h	7.25	1.09, 205	0.03	0.2
Age	0.60	0.08, 1.57	0.3	
Gender	1.34	0.10, 21.8	0.8	

Abbreviation: CI = confidence interval.

**Table 3:** Regression models for D-DT levels predicting right heart failure (RHF). Predictive Firth Regression models demonstrate that D-DT levels did not independently predict post-OP RHF at either time point.

Predictive Firth Regression Models for Right Heart Failure				
Characteristic	Exp(Beta)	95% CI	*p*-Value	q (FDR)
Covariates only				
Age	0.96	0.32, 2.21	>0.9	
Gender	3.01	0.44, 32.1	0.3	
Preoperative D-DT levels				
D-DT pre-OP	0.62	0.21, 1.65	0.3	0.5
Age	1.16	0.40, 2.89	0.7	
Gender	4.34	0.50, 77.3	0.2	
Postoperative D-DT levels				
D-DT post-OP	0.97	0.35, 2.68	>0.9	>0.9
Age	0.88	0.26, 2.37	0.8	
Gender	2.55	0.37, 27.0	0.3	
24 h D-DT levels				
D-DT 24 h	2.36	0.92, 10.2	0.077	0.2
Age	1.18	0.31, 3.26	0.8	
Gender	4.35	0.60, 54.8	0.15	

Abbreviation: CI = Confidence Interval.

## Data Availability

The data of this study are available upon reasonable request from the corresponding author.

## Supplementary Materials

The following supporting information can be downloaded at: https://www.mdpi.com/article/10.3390/biomedicines14051031/s1, Table S1: Discriminatory power of single analytes and their ratios with sCD74 across the different time points; Figure S1: Forest plots of the Interaction firth regression models of sCD74/MIF ratio and D-DT levels for composite outcome and right heart failure (RHF), respectively; Figure S2: Kaplan–Meier graph of probability of remaining in ICU and detailed information about the statistical methodology; Table S2: rMIF/sCD74 reduces absolute cell counts after 48 h; Table S3: rD-DT and rD-DT/sCD74 reduce absolute cell counts after 48 h; Table S4: Descriptive statistics including Bonferroni correction of Alamar Blue assay; Table S5: Descriptive statistics and 2-way-ANOVA of Annexin V/PI Flow cytometry; Table S6: Descriptive statistics including Bonferroni correction of BrdU-incorporation assay; Table S7: Fold change in inflammatory genes; Table S8: TaqMan probes with Assay IDs.

## Abbreviations

The following abbreviations are used in this manuscript:

MIF	macrophage migration inhibitory factor
rMIF	recombinant MIF
D-DT	D-Dopachrome tautomerase
rD-DT	recombinant D-DT
sCD74	soluble cluster of differentiation 74
ESHF	end-stage heart failure
CMF	cardiac myofibroblasts
LVAD	left ventricular assist device
CVD	cardiovascular disease
CHD	coronary heart disease
MI	myocardial infarction
HF	heart failure
CM	cardiomyocytes
CF	cardiac fibroblasts
MHC class II	major histocompatibility complex class II
CD44	cluster of differentiation 44
CXCR	CXC-motif receptor
AKI	acute kidney injury
α-SMA	alpha smooth muscle actinin
PBS	phosphate buffered saline
BSA	bovine serum albumin
FACS	fluorescence automated cell sorting
BrdU	Bromodeoxyuridine
ELISA	enzyme linked immunosorbent assay
RHF	right heart failure
DAPI	4′,6-diamidino-2-phenylindole
ROC	receiver operating characteristics
CI	confidence interval
ICU	intensive care unit
COPD	chronic obstructive pulmonary disease
CPB	cardiopulmonary bypass
IFN	interferon
AKT	protein kinase B/AKT-kinase
