# Supplementary material for: Circulating MIF, D-DT, and Soluble CD74 in End-Stage Heart Failure Patients Receiving LVAD: An Exploratory Clinical Study and Effects on Adult Cardiac Myofibroblasts
Source: Biomedicines. 2026 Apr 30;14(5):1031. doi: 10.3390/biomedicines14051031 (PMC13203901; doi:10.3390/biomedicines14051031)
Supplement: Supplementary file 1 [file biomedicines-14-01031-s001.zip › biomedicines-4245754-supplementary.pdf]

Supplemental Material

Circulating MIF, D-DT, and soluble CD74 in end-stage heart failure patients receiving LVAD: An exploratory clinical study and effects on adult cardiac myofibroblasts

M. Kunze[1], M. Uhlig[1], A. Theißen[1], C. Stoppe[2,3,4,5*], C. Beckers[1], J. Larmann[1], R. Zayat[6], A. Moza[6], J. Bernhagen[7,8,9], A. Goetzenich[6,10], C. Bleilevens[1], J. Soppert[1,6,*]

1 Department of Anesthesiology, Faculty of Medicine, University Hospital RWTH Aachen, Aachen, Germany
2 Department of Anesthesiology, Intensive Care, Emergency and Pain Medicine, University Hospital Würzburg, Würzburg, Germany
3 Department of Cardiac Anesthesiology and Intensive Care Medicine, Deutsches Herzzentrum der Charité, Berlin, Germany
4 Charité-Universitätsmedizin Berlin, Corporate Member of Freie Universität Berlin, Humboldt-Universität zu Berlin, Berlin, Germany
5 DZHK (German Center for Cardiovascular Research), partner site Berlin, Berlin, Germany
6 Department of Cardiac Surgery, Faculty of Medicine, University Hospital RWTH Aachen, Aachen, Germany
7 Division of Vascular Biology, Institute for Stroke and Dementia Research (ISD), LMU Klinikum, Ludwig Maximilian University (LMU) Munich, Munich, Germany
8 German Centre for Cardiovascular Research (DZHK), partner site Munich Heart Alliance, Munich, Germany
9 Munich Cluster for Systems Neurology (SyNergy), Munich, Germany
10 Medical Affairs, Abiomed GmbH, 52074 Aachen, Germany
***** Correspondence: JS - jsoppert@ukaachen.de; +49 242 80 38221; CS - christian.stoppe@gmail.com

Exploratory retrospective clinical data

***Table S1: Discriminatory power of single analytes and their ratios with sCD74 across the different time points**. Area under the receiver operating characteristic (ROC) curve by simple logistic regression models for the primary composite outcome, defined as early death within the first 30 days or prolonged stay at the intensive care unit with at least 20 days. ROC curves for postoperative complications, such as acute kidney injury, pneumonia, right heart failure and rethoracotomy. AUC, area under the curve; CI, confidence interval.*

	AUC	Standard Error	95 % CI			p-value
**Composite outcome**						
MIF (post-OP)	0.53	0.139	0.26	-	0.81	0.81
MIF (24 hours)	0.64	0.130	0.38	-	0.89	0.31
sCD74 (post-OP)	0.53	0.141	0.26	-	0.81	0.81
sCD74 (24 hours)	0.74	0.119	0.50	-	0.97	0.074
D-DT (post-OP)	0.53	0.139	0.26	-	0.81	0.81
D-DT (24 hours)	0.71	0.121	0.47	-	0.94	0.12
sCD74/MIF (post-OP)	0.53	0.140	0.26	-	0.81	0.81
**sCD74/MIF (24 hours)**	**0.82**	**0.096**	**0.63**	**-**	**1.00**	**0.017**
sCD74/D-DT ratio (post-OP)	0.53	0.138	0.26	-	0.80	0.81
sCD74/D-DT ratio (24 hours)	0.61	0.139	0.33	-	0.88	0.43
**Acute kidney injury**						
MIF (post-OP)	0.77	0.116	0.54	-	0.99	0.05
MIF (24 hours)	0.53	0.136	0.26	-	0.80	0.82
sCD74 (post-OP)	0.62	0.135	0.36	-	0.89	0.37
**sCD74 (24 hours)**	**0.83**	**0.093**	**0.65**	**-**	**1.00**	**0.013**
D-DT (post-OP)	0.54	0.136	0.28	-	0.81	0.74
D-DT (24 hours)	0.66	0.127	0.41	-	0.81	0.23
sCD74/MIF (post-OP)	0.70	0.124	0.46	-	0.94	0.14
sCD74/MIF (24 hours)	0.67	0.131	0.41	-	0.93	0.2
sCD74/D-DT ratio (post-OP)	0.52	0.141	0.25	-	0.80	0.87
sCD74/D-DT ratio (24 hours)	0.53	0.137	0.26	-	0.79	0.85
**Pneumonia**						
MIF (post-OP)	0.66	0.140	0.38	-	0.93	0.31
MIF (24 hours)	0.54	0.138	0.26	-	0.81	0.81
sCD74 (post-OP)	0.53	0.184	0.17	-	0.89	0.85
sCD74 (24 hours)	0.54	0.143	0.26	-	0.82	0.81
D-DT (post-OP)	0.63	0.148	0.34	-	0.92	0.41
D-DT (24 hours)	0.57	0.165	0.25	-	0.90	0.62
sCD74/MIF (post-OP)	0.63	0.130	0.37	-	0.88	0.41
sCD74/MIF (24 hours)	0.57	0.131	0.32	-	0.83	0.62
sCD74/D-DT ratio (post-OP)	0.66	0.154	0.35	-	0.96	0.31
sCD74/D-DT ratio (24 hours)	0.51	0.134	0.24	-	0.77	0.97
**Right heart failure**						
MIF (post-OP)	0.63	0.150	0.33	-	0.92	0.38
MIF (24 hours)	0.69	0.136	0.42	-	0.96	0.19
sCD74 (post-OP)	0.52	0.158	0.21	-	0.83	0.87
sCD74 (24-hours)	0.65	0.127	0.41	-	0.90	0.28
**D-DT (post-OP)**	**0.83**	**0.115**	**0.61**	**-**	**1.00**	**0.023**
**D-DT (24 hours)**	**0.88**	**0.112**	**0.66**	**-**	**1.00**	**0.008**
sCD74/MIF (post-OP)	0.60	0.149	0.31	-	0.90	0.48
sCD74/MIF (24 hours)	0.79	0.108	0.57	-	0.9968	0.048
**sCD74/D-DT ratio (post-OP)**	**0.83**	**0.104**	**0.63**	**-**	**1.00**	**0.023**
**sCD74/D-DT ratio (24 hours)**	**0.87**	**0.103**	**0.67**	**-**	**1.00**	**0.011**
**Rethoracotomy**						
MIF (post-OP)	0.67	0.128	0.42	-	0.92	0.24
MIF (24 hours)	0.58	0.135	0.32	-	0.85	0.55
sCD74 (post-OP)	0.52	0.158	0.21	-	0.83	0.87
sCD74 (24 hours)	0.60	0.141	0.33	-	0.88	0.45
D-DT (post-OP)	0.54	0.146	0.25	-	0.82	0.8
D-DT (24 hours)	0.63	0.133	0.37	-	0.89	0.36
sCD74/MIF (post-OP)	0.69	0.128	0.44	-	0.94	0.18
sCD74/MIF (24 hours)	0.62	0.139	0.34	-	0.89	0.41
sCD74/D-DT ratio (post-OP)	0.56	0.141	0.28	-	0.84	0.67
sCD74/D-DT ratio (24 hours)	0.54	0.145	0.26	-	0.83	0.75

Detailed statistical analysis

To evaluate the association between the sCD74/MIF ratio and the composite outcome, Firth's penalized likelihood logistic regression was applied using the logistf package (Version 1.26.1). This approach was selected due to its robustness in the presence of small sample sizes and to address issues of quasi-complete separation, where conventional logistic regression fails to converge.

Three measurement timepoints were considered: preoperative baseline (pre OP), immediately postoperative (post OP) and 24 hours after surgery (24 hours). In further analyses we concentrated on the latter regarding the results of Firth's penalized likelihood logistic regression.

To evaluate the discriminatory power of each analyte as well as their ratios for each time point, receiver operating characteristic (ROC) analysis were performed using GraphPad Prism (version 10.4.0, GraphPad Software, Inc., La Jolla, CA, USA), calculating the area under the curve (AUC) and its 95 % confidence interval to assess overall discriminative performance. To identify a clinically relevant threshold, the optimal cutpoint was determined using the cutpointr package (version 1.2.1). The cutpoint was selected by maximizing the Youden Index (sensitivity + specificity − 1), a standard approach for identifying the best-performing binary classification threshold.

To visualize and quantify the association between preoperative sCD74 levels and ICU stay duration, patients were stratified into two groups based on the previously identified optimal cutpoint (sCD74/MIF ratio at 24 hours > 3.817). Kaplan-Meier curves were generated using the survminer (version 0.5.0) and survival (version 3.8.3) packages to visualize the probability of remaining in the ICU over time for each group. Differences in ICU discharge rates were further analyzed using a Cox proportional hazards regression model, adjusting for relevant covariates (age and gender). The resulting odds ratio (OR) was interpreted as the relative likelihood of remaining in ICU, with an HR > 1 indicating a longer stay in the high-risk group (sCD74/MIF ratio at 24 hours > 3.817). $P < 0.05$ was considered as significant.

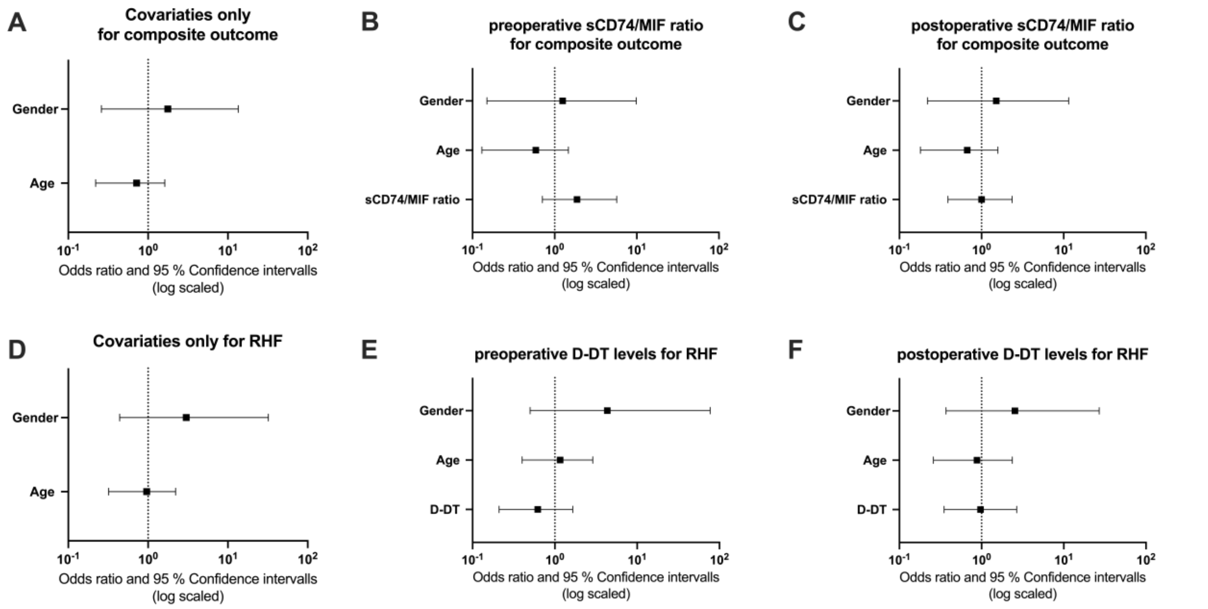

Figure S1: Forest plots of the Interaction firth regression models of sCD74/MIF ratio and D-DT levels for composite outcome and right heart failure (RHF), respectively. ***(A-C)*** *For composite outcome, neither covariates independently* ***A*** *or sCD74/MIF with or without adjustments for covariates preoperative* ***B*** *and postoperative* ***C*** *can be correlated with the composite outcome.* ***(D-F)*** *For RHF, neither covariates independently* ***D*** *or D-DT with or without adjustments for covariates preoperative* ***E*** *and postoperative* ***F*** *can be correlated with the RHF.*

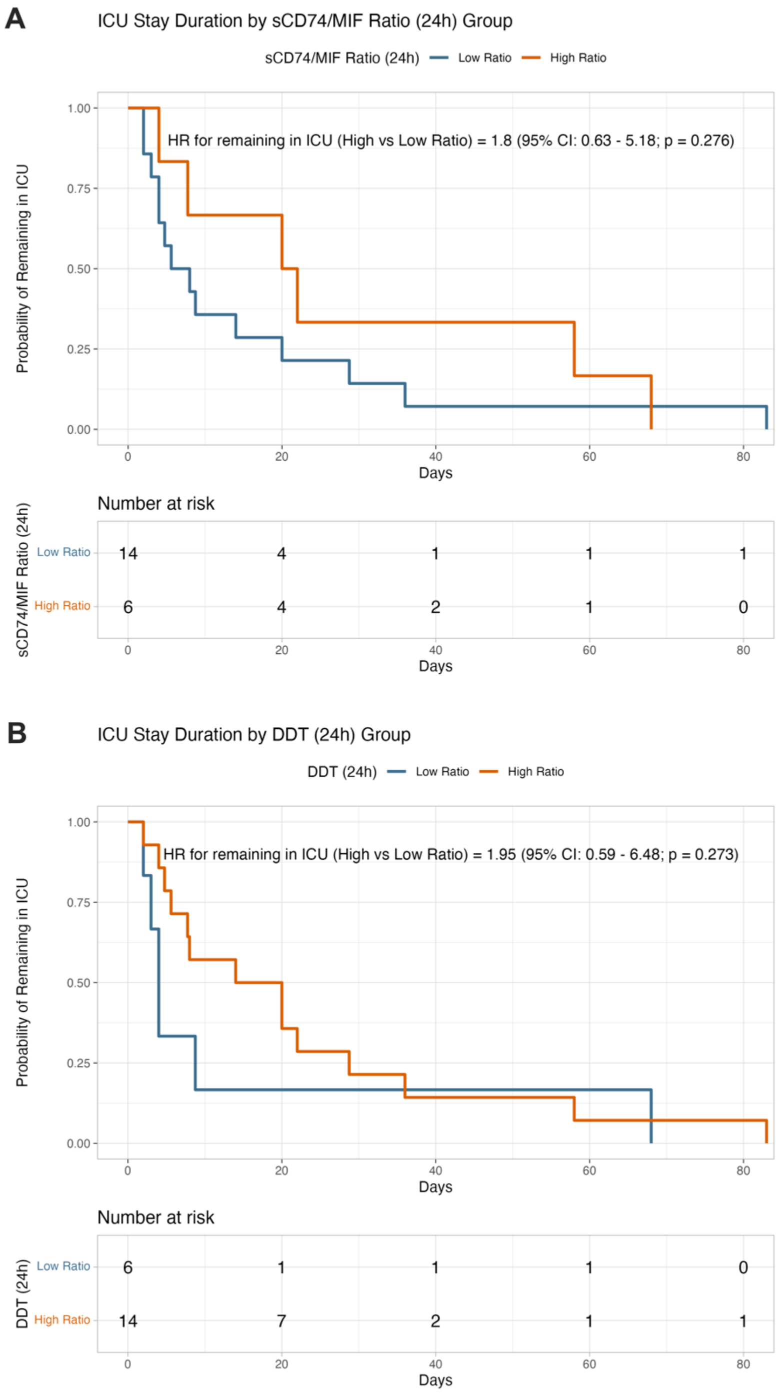

Figure S2: ***Kaplan-Meier graph of probability of remaining in ICU. A*** *Patients with higher sCD74/MIF ratio 24 hours post-surgery (> 3.817) show a tendency, but not significant, to longer ICU stays compared to low ratios (Adjusted hazard ratio (HR) = 1.8, 95 % CI: 0.63 – 5.18, p = 0.276).* ***B*** *Patients with higher D-DT levels 24 hours post-surgery (> 3.817) show a tendency, but not significant, to longer ICU stays compared to low ratios (Adjusted hazard ratio (HR) = 1.95, 95 % CI: 0.59 – 6.48, p = 0.273). p < 0.05 is considered significant.*

Experimental cell culture model of adult cardiac fibroblasts

Trypan Blue exclusion assay

Table S2: rMIF/sCD74 reduces absolute cell counts after 48 hours *– Absolute total and viable counts after stimulation with rMIF, sCD74 or rMIF/sCD74 were compared to control using one-way ANOVA with Tukey´s multiple comparison test. p-value < 0.05 is considered significant.*

Tukey's multiple comparisons test	Mean difference	95 % confidence interval	Adjusted p-value
Total cell count (x 10^5)			
Control vs. rMIF	0.2543	-0.3834, 0.8920	0.80
Control vs. sCD74	0.4354	-0.2023, 1.073	0.32
Control vs. rMIF/sCD74	0.8372	0.1995, 1.475	0.0040
Viable cell count (x 10^5)			
Control vs. rMIF	0.2264	-0.4146, 0.8675	0.86
Control vs. sCD74	0.3832	-0.2579, 1.024	0.46
Control vs. rMIF/sCD74	0.7879	0.1469, 1.429	0.0083

Table S3: rD-DT and rD-DT/sCD74 reduce absolute cell counts after 48 hours *– Absolute total and viable counts after stimulation with rD-DT, sCD74 or rD-DT/sCD74 were compared to control using one-way ANOVA with Tukey´s multiple comparison test. p-value < 0.05 is considered significant.*

**Tukey's multiple comparisons test**	**Mean difference**	**95 % confidence interval**	**Adjusted p-value**
Total cell count (x 10^5)			
Control vs. rD-DT	0.9371	0.1800, 1.694	0.0077
Control vs. sCD74	0.8737	0.1166, 1.631	0.016
Control vs. rD-DT/ sCD74	1.177	0.4197, 1.934	0.0004
Viable cell count (x 10^5)			
Control vs. rD-DT	0.9950	0.2578, 1.732	0.0028
Control vs. sCD74	0.8123	0.07505, 1.550	0.024
Control vs. rD-DT/ sCD74	1.240	0.5024, 1.977	0.0001

Alamar Blue metabolic assay

*Table S4: **Descriptive statistics including Bonferroni correction of Alamar Blue assay** – Adult cardiac fibroblast were stimulated with rMIF, sCD74 and a combination of both for 48 hours. Alamar Blue reduction has been assessed as measure of metabolic activity at four and eight hours after Alamar Blue incubation. Statistical significance was assessed by normalizing absorption values to control wells (set to 100%) and applying a one-sample t-test, followed by Bonferroni post hoc correction. p-value < 0.05 is considered significant.*

	4 hours incubation time			8 hours incubation time		
	**rMIF**	**sCD74**	**rMIF/sCD74**	**rMIF**	**sCD74**	**rMIF/sCD74**
Theoretical mean	100	100	100	100	100	100
Actual mean	98.94	97.56	98.3	100.4	98.89	99.01
Standard deviation	4.16	5.62	4.71	6.83	7.32	4.61
Number of values	4	4	4	4	4	4
**One sample t-test**						
t, df	t=0.5098, df=3	t=0.8667, df=3	t=0.7213, df=3	t=0.1055, df=3	t=0.3043, df=3	t=0.4281, df=3
P-value (two tailed)	0.6453	0.4499	0.5229	0.9227	0.7808	0.6974
Bonferroni correction (3 comparisons)	1	1	1	1	1	1
P value summary	ns	ns	ns	ns	ns	ns
Significance (alpha ≤ 0.05)	no	no	no	no	no	no

The Percentage difference between treatment groups in the Alamar blue assay was calculated using the following calculation:

$$Percentage\ difference\ in\ reduction = \frac{A_{LW} - (A_{HW} * R_0)\ for\ test\ well}{A_{LW} - (A_{HW} * R_0) for\ control\ well} * 100$$

A(LW)= absorbance of lower wavelength minus absorbance in media blank

A(HW)= absorbance of higher wavelength minus absorbance in media blank

Source: Bio-Rad (https://www.bio-rad-antibodies.com/spectrophotometry-calculations-alamarblue-different-filters.html)

Annexin V/PI flow cytometry

Table S5: Descriptive statistics and 2-way-ANOVA of Annexin V/PI Flow cytometry *– To assess different types of cell death and their proportion Annexin V/PI staining and flowcytometry was performed. Cardiac fibroblasts were cultured, stimulated, detached and stained according to protocol. 100'000 events were recorded and data is presented in percentage of total cell count. Treatment groups were compared to control by 2-way-ANOVA with Bonferroni correction. p-value < 0.05 is considered significant.*

	**viable cells**			**early apoptotic cells**			**necrotic cells**			**late apoptotic cells**		
	**Mean**	**SD**	**p-value**	**Mean**	**SD**	**p-value**	**Mean**	**SD**	**p-value**	**Mean**	**SD**	**p-value**
Control	94,93	0,83		0,30	0,00		3,50	0,26		1,33	0,58	
rMIF	94,97	1,10	>0,99	0,43	0,06	>0,99	3,07	0,38	>0,99	1,53	0,78	>0,99
sCD74	93,80	1,93	>0,99	0,50	0,10	>0,99	3,97	1,38	>0,99	1,70	0,89	>0,99
rMIF/ sCD74	94,70	1,37	>0,99	0,33	0,06	>0,99	3,53	1,29	>0,99	1,43	0,25	>0,99
rD-DT	94,37	1,27	>0,99	0,53	0,15	>0,99	3,67	1,07	>0,99	1,43	0,15	>0,99
rD-DT/ sCD74	93,80	0,36	>0,99	0,43	0,15	>0,99	3,83	0,32	>0,99	1,90	0,56	>0,99

BrdU-incorporation assay

Table S6: Descriptive statistics including Bonferroni correction of BrdU-incorporation assay *– To analyze cell proliferation an BrdU- incorporation was used. Cells were stimulated with rMIF, sCD74, rD-DT and combinations of rMIF/sCD74 or rD-DT/sCD74 for 48 hours with the addition of BrdU for the last 16 hours. Statistical significance was assessed by normalizing absorption values to control wells (set to 100%) and applying a one-sample t-test, followed by Bonferroni post hoc correction. p-value < 0.05 is considered significant. No significant differences the groups compared to control could be shown.*

	**rMIF**	**sCD74**	**rMIF/sCD74**	**rD-DT**	**rD-DT/sCD74**
Theoretical mean	100	100	100	100	100
Actual mean	96.01	98.68	92.49	96.27	97.6
Standard deviation	9.13	7.46	12.33	10.02	7.80
Number of values	3	3	3	3	3
**One sample t-test**					
t, df	t=0.7566, df=2	t=0.3064, df=2	t=1.055, df=2	t=0.6449, df=2	t=0.5340, df=2
P-value (two tailed)	0.5283	0.7883	0.4021	0.5851	0.6467
Bonferroni correction (3 comparisons)	1	1	1	1	1
P value summary	ns	ns	ns	ns	ns
Significance (alpha ≤ 0.05)	no	no	no	no	no

Quantitative real time PCR

Table S7: Fold change of inflammatory genes *- Cardiac fibroblast were cultured and stimulated according to protocol. After 16 hours cells were lysated. RNA isolated and transformed into dsDNA. Three independent experiments were run. Fold change is presented as mean ± SD. P-values were calculated using One-way ANOVA with Dunnett correction. P < 0.05 was considered significant. Clec4e and Irg1 could not be detected under any condition. Actb.- actin beta; GAPDH – glycerinaldehyde-3-phosphate dehydrogenase; Ccl – C-C motif chemokine ligand; Clec4e – C-type lectin domain family 4 member e; Ifi44 – interferon-induced protein 44; Irg1 – immunoresponsive gene 1; Oasl2 – 2'-5' oligoadenylate synthetase-like 2; Isg15 – interferon stimulated gene 15*

Gene	**Stimulus**	**mean fold change (RQ)**	**± SD**	**p-value**
Actb	rMIF	1.082	0.1586	0.9606
	sCD74	1.069	0.1631	0.9811
	rMIF/sCD74	1.07	0.2666	0.9795
	rD-DT	1.196	0.2007	0.5119
	rD-DT/sCD74	1.032	0.09991	0.9994
Ccl2	rMIF	1.084	0.2256	0.9887
	sCD74	1.377	0.4345	0.2371
	rMIF/sCD74	1.104	0.1764	0.9728
	D-DT	1.142	0.1694	0.9108
	D-DT/sCD74	1.361	0.1757	0.2697
Ccl7	rMIF	0.6523	0.09023	0.0741
	sCD74	1.192	0.2655	0.4718
	rMIF/sCD74	0.9607	0.1972	0.9979
	rD-DT	0.8503	0.1503	0.6782
	rD-DT/sCD74	1.057	0.1092	0.9884
Ifi44	rMIF	1.239	0.534	0.9974
	sCD74	1.911	1.95	0.643
	rMIF/sCD74	1.068	0.5929	>0.9999
	rD-DT	1.261	0.5232	0.9961
	rD-DT/sCD74	1.322	0.6563	0.9901
Oasl2	rMIF	1.33	0.4415	0.9142
	sCD74	1.482	0.9511	0.7302
	rMIF/sCD74	1.192	0.519	0.99
	rD-DT	1.357	0.5417	0.8879
	rD-DT/sCD74	1.409	0.3974	0.8283
Isg15	rMIF	1.4	0.6469	0.9474
	sCD74	1.829	1.375	0.5647
	rMIF/sCD74	1.075	0.5461	>0.9999
	rD-DT	1.401	0.882	0.9471
	rD-DT/sCD74	1.517	0.3576	0.8713

Table S8: TaqMan probes with Assay IDs - Actb.- actin beta; GAPDH – glycerinaldehyde-3-phosphate dehydrogenase; Ccl – C-C motif chemokine ligand; Clec4e – C-type lectin domain family 4 member e; Ifi44 – interferon-induced protein 44; Irg1 – immunoresponsive gene 1; Oasl2 – 2'-5' oligoadenylate synthetase-like 2; Isg15 – interferon stimulated gene 15

TaqMan probe/ Gene	Assay ID
Actb	Mm02619580_g1
Gapdh	Mm99999915_g1
Ccl2	Mm00441242_m1
Ccl7	Mm00443113_m1
Clec4e	Mm01183703_m1
Ifi44	Mm00505670_m1
Irg1	Mm01224532_m1
Oasl2	Mm01201449_m1
Isg15	Mm01705338_s1
